# Unexpected Exacerbation of Tracheal Stenosis in a Patient with Hunter Syndrome Undergoing Cardiac Surgery

**DOI:** 10.1155/2018/5691410

**Published:** 2018-05-10

**Authors:** Nobue Terabe, Soichiro Yamashita, Makoto Tanaka

**Affiliations:** ^1^Department of Anesthesiology, University of Tsukuba Hospital, Tsukuba, Japan; ^2^Department of Anesthesiology, Faculty of Medicine, University of Tsukuba, Tsukuba, Japan

## Abstract

We report unexpected exacerbation of tracheal stenosis during general anesthesia in a 50-year-old patient with Hunter syndrome undergoing cardiac surgery for valvular disease. He had undergone cervical laminoplasty 3 months previously; at that time, his airway had been uneventfully managed. Preoperative flexible fiberoptic laryngoscopy showed a normal upper respiratory tract, but chest computed tomography showed tracheal stenosis that had flattened the lumen. The narrowest part above the tracheal bifurcation was 2 cm long and the anteroposterior diameter was ≤6 mm. Cardiac surgery was uneventfully performed. After weaning from cardiopulmonary bypass, the tidal volume suddenly decreased from 450 to 120 ml at sternal closure. The end-expiratory carbon dioxide pressure increased from 39 to 71 mmHg. Bronchoscopic examination showed that the part of tracheal bifurcation was almost occluded. A tidal volume of 400 ml was obtained after the transesophageal echocardiography probe was removed and the peak inspiratory pressure increased. Although extubation was performed on the second postoperative day, procaterol inhalation and noninvasive positive-pressure ventilation were needed for 3 days because of wheezing and dyspnea. In conclusion, the risk of lower respiratory tract obstruction should be considered during general anesthesia in patients with Hunter syndrome with collapsible tracheal stenosis undergoing cardiac surgery.

## 1. Introduction

Hunter syndrome is characterized by a deficiency of the iduronate-2-sulfatase required for mucopolysaccharide degradation [1]. Various complications should be considered in patients with Hunter syndrome undergoing surgery because intracellular accumulation of glycosaminoglycans (GAGs) causes progressive damage to various organs and tissues [2, 3]. We herein report unexpected exacerbations of tracheal stenosis during general anesthesia in an adult patient with Hunter syndrome undergoing cardiac surgery and discuss some problems derived from pathological changes in the lower respiratory tract. Written informed consent was obtained from the patient for publication of this case report and accompanying images.

## 2. Case Presentation

**Figure 1:**
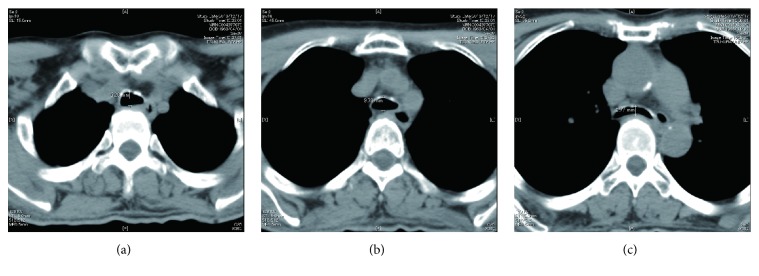
Chest computed tomography 1.5 months previously. (a) The anteroposterior diameter of the trachea at 3 cm away from the glottis was 10.3 mm. (b) The anteroposterior diameter of the trachea at 6 cm away from the glottis was 9.8 mm. (c) The anteroposterior diameter of the tracheal bifurcation at 9 cm away from the glottis was 5.8 mm.

A 50-year-old, 50-kg, 152-cm man with aortic and mitral valvular disease was scheduled for a double valve replacement. He had been diagnosed with Hunter syndrome at the age of 40 years and had received enzyme replacement therapy with idursulfase infusion. He had undergone general anesthesia 3 months previously for cervical laminoplasty at another hospital, and his airway had been managed uneventfully at that time with a reinforced tracheal tube of 7-mm inner diameter (ID). He showed the distinctive features of a short neck, jaw deformation, and macroglossia, and he had slight cervical motor restriction and a Mallampati classification of Ш. Preoperative flexible fiberoptic laryngoscopy 2 week previously showed that the patency of the upper respiratory tract was kept though oropharyngeal soft tissue thickening was recognized. However, chest computed tomography (CT) 1.5 months previously showed tracheal stenosis that had flattened the lumen (Figure 1). The narrowest part above the tracheal bifurcation was 2 cm long and the anteroposterior diameter was ≤6 mm, but the patency of the upper part of the trachea was adequate for insertion of a tracheal tube of 7-mm ID. Cardiac ultrasound demonstrated severe aortic valve stenosis, moderate aortic valve regurgitation, and mild mitral valve regurgitation. The aortic valve area was 0.53 cm^2^. The maximum and mean aortic valve pressure gradients were 106 and 67 mmHg, respectively. The left ventricle was generally enlarged and the ejection fraction was 71% (Simpson's method). Abdominal CT showed hepatosplenomegaly. All blood parameters were within the normal ranges. During a preoperative conference, we assessed that his airway could be managed in the same way as during the previous anesthetic procedure because the patient had not felt difficulty in breathing.

**Figure 2:**
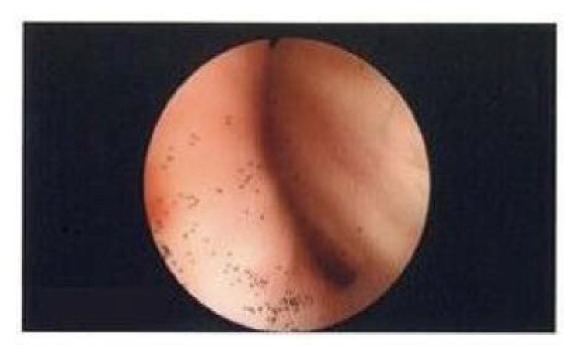
Bronchoscopy after induction of anesthesia revealed tracheal stenosis that gradually became more severe from under the glottis.

At the time of admission, his blood pressure was 139/47 mmHg, heart rate was 65 beats/min, and peripheral oxygen saturation was 97%. After cannulation to the left radial artery for continuous monitoring of arterial blood pressure, 3 mg of midazolam and 200 *μ*g of fentanyl were administered for induction of anesthesia. After confirming mask ventilation, 60 mg of rocuronium bromide was provided for neuromuscular blockade. The glottis was easily confirmed using the Airway Scope (Pentax-AWS®; Hoya, Tokyo, Japan), and a tracheal tube of 7-mm ID was inserted into the trachea. However, the tube could not be advanced more than 3 cm from the glottis. The tube was removed. The upper airway was then secured using an i-gel #4 (Nihon Kohden, Tokyo, Japan), and bronchoscopic examination was performed through the breathing channel of the i-gel. Tracheal stenosis that gradually became more severe from under the glottis was revealed (Figure 2). Therefore, a tracheal tube of 6-mm ID was inserted using the Airway Scope. We felt slight resistance during insertion of the tube, but an audible leak around the tube with an inspiratory pressure of 20 cmH_2_O was heard when the cuff was deflated. The tube was secured at the 22-cm mark at the alveolar ridge. Pressure-controlled ventilation was started with a peak inspiratory pressure of 20 cmH_2_O and a respiratory rate of 12 breaths/min, and a tidal volume of about 450 ml was obtained. Although a transesophageal echocardiography (TEE) probe was inserted, the tidal volume did not change.

The aortic and mitral valve replacement was uneventfully performed under cardiopulmonary bypass. After aortic declamping, the patient was easily weaned from cardiopulmonary bypass with a 5-*μ*g/kg/min dopamine infusion. Cardiac function after weaning was good. However, the tidal volume suddenly decreased from 450 to 120 ml at sternal closure, and the end-expiratory carbon dioxide pressure increased from 39 to 71 mmHg. Bronchoscopic examination showed that the stenosis became more severe than that after induction of anesthesia, and the part of the tracheal bifurcation was almost occluded. The tidal volume was hardly obtained in spite of a peak inspiratory pressure of >30 cmH_2_O. Therefore, we tried to remove the TEE probe because it might have been exacerbating the tracheal stenosis by pushing the trachea adjacent to the esophagus. As a result, a tidal volume of 400 ml was obtained with a peak inspiratory pressure of 25 cmH_2_O by improving the severe stenosis of the tracheal bifurcation, and the end-expiratory carbon dioxide pressure decreased to 43 mmHg. In addition, 125 mg of methylprednisolone was administered intravenously to reduce edema in the lower respiratory tract. Extubation was performed on the second postoperative day. After extubation, the patient's oxygenation was good but wheezing and dyspnea were noted. Procaterol inhalation and noninvasive positive-pressure ventilation were administered for 3 days. After his breathing had become stable, the patient was discharged from the hospital on the 25th postoperative day.

## 3. Discussion

Intracellular accumulation of GAGs causes progressive damage to various organs and tissues. Although the extent of organ damage and disease progression are variable, approximately 75% of patients with Hunter syndrome are severely affected and die in the first or second decade of life. The remaining patients have normal cognitive function and survive into adulthood [2]. Valvular disease is frequent in patients with Hunter syndrome but cardiac surgery remains uncommon because of the short lifespan of severely affected patients [4–6]. However, various complications due to GAGs accumulation should be considered in patients with Hunter syndrome undergoing cardiac surgery [2, 3]. In the present case, we encountered unexpected exacerbation of tracheal stenosis at sternal closure after weaning from cardiopulmonary bypass. This critical episode highlights the risk of lower respiratory tract obstruction during general anesthesia in patients with Hunter syndrome undergoing cardiac surgery.

Accumulation of GAGs in the lower respiratory tract causes softening and weakness of the supporting cartilage, resulting in tracheobronchial stenosis and malacia [7]. In the present case, the trachea had already been affected by malacia because tracheal stenosis that had flattened the lumen was observed on the preoperative CT. In such a condition, the trachea might be collapsed by increased intramediastinal pressure at sternal closure. Patients with Hunter syndrome have several factors which might contribute to the lower respiratory tract obstruction caused by this mechanism. Myocardial edema and increased intraventricular filling pressure due to volume load after cardiopulmonary bypass might increase the intramediastinal pressure because the hypertrophic myocardium had been already occupying the intramediastinal space [8]. Stiffness of the chest wall and reduced thoracic volume due to hepatosplenomegaly might also enhance the intramediastinal pressure [2, 3]. In addition, mucosal edema related to systemic inflammation due to cardiopulmonary bypass might contribute to exacerbation of the tracheal stenosis because of mucosal thickening [2, 9]. Increased sputum production due to reduced mucociliary clearance might increase the airway resistance [10]. Moreover, TEE probe might also contribute to exacerbation of the tracheal stenosis by pushing the collapsible trachea [11]. Therefore, we must consider that patients with Hunter syndrome with collapsible tracheal stenosis have the risk of lower respiratory tract obstruction during cardiac surgery. Checking the change in airway diameter during respiration in dynamic chest CT may be useful to predict the collapsibility of the tracheobronchial wall [10].

On the other hand, a tracheal tube of the same size as that used during general anesthesia only 3 months previously could not be advanced more than 3 cm from the glottis after induction of anesthesia. Although the patency of the upper part of the trachea was still adequate for insertion of a tracheal tube of 7-mm ID based on the preoperative CT performed 1.5 months previously, the tracheal stenosis gradually progressed. Increased intramediastinal pressure by cardiac dilation due to valvular disease might contribute to exacerbation of the tracheal stenosis because the trachea had already been affected by malacia. Another possible explanation is that mechanical stimulation by the tracheal tube that had been used during the recent general anesthetic procedure 3 months previously might have contributed to exacerbation of the tracheal stenosis. A previous report showed that any instrumentation of the airway in patients with Hunter syndrome might induce increased mucopolysaccharide deposition and that the mucosal injury and ischemia caused by advancing the tube or inflating the cuff might lead to disease progression [12]. However, the speed of disease progression is definitively unknown. In the review article, the authors describe the fact that the interval between multiple planned surgical procedures should be sufficiently short because of the progressive nature of Hunter syndrome [2]. Therefore, the possibility of disease progression in the lower respiratory tract should be considered even if the airway was uneventfully managed during a recent general anesthetic procedure in patients with Hunter syndrome, and a smaller tracheal tube should be selected.

The anesthetic management of patients with tracheal stenosis is one of the most clinical challenges for the anesthesiologist [13, 14]. If patients have critical airway obstruction, maintenance of spontaneous ventilation is theoretically important as conversion to positive-pressure ventilation can lead to an aggravation of the obstruction or complete airway collapse. In those patients, an inhalational anesthetic agent (sevoflurane) which maintains spontaneous ventilation is preferred over intravenous anesthetic agents for induction of anesthesia. Otherwise, an awake intubation under sedation or rapid induction with using an extracorporeal membrane oxygenator can be selected according to the risk of airway collapse [3]. In the present case, a conventional rapid induction was performed because the patient had not felt difficulty in breathing. A muscle relaxant was administrated after checking whether a trachea stenosis was worsened by a cessation of the spontaneous ventilation.

I-gel is designed for airway maintenance during general anesthesia and is also useful as a rescue device in unpredicted difficult airway [15]. The conduit of i-gel facilitates the fiberoptic examination of the vocal cord and trachea while improving oxygenation by intermittent ventilation and reducing our stress associated with difficult airway situations. Because wide bore of the conduit allows passage of a tracheal tube, fiberoptic-guided tracheal intubation is possible if necessary.

Various anesthetic risks other than tracheobronchial stenosis and malacia should also be considered during general anesthesia in patients with Hunter syndrome [2, 3]. GAGs accumulations in the mucosa and soft tissues of the upper respiratory tract cause enlargement of the larynx, tonsils, adenoids, and tongue, leading to a difficult airway or obstruction during general anesthesia [16, 17]. Spinal cord compression may occur due to spinal canal narrowing. A short neck and reduced cervical joint mobility also contribute to a difficult airway. Restrictive pulmonary disease can develop due to thoracic-cage abnormalities or compromised excursion of the diaphragm secondary to an enlarged liver and spleen. Cardiac risks other than valvular disease include diastolic dysfunction from the hypertrophied myocardium, or complete atrioventricular block.

## 4. Conclusion

Patients with Hunter syndrome with collapsible tracheal stenosis have the risk of lower respiratory tract obstruction during cardiac surgery. Careful airway evaluation and management are required while considering the pathological changes of the lower respiratory tract in patients with Hunter syndrome undergoing cardiac surgery.
